# The NMR-measured omega-6/omega-3 fatty acid ratio improves cardiovascular risk prediction

**DOI:** 10.3389/fnut.2025.1693151

**Published:** 2025-10-29

**Authors:** Youwei Huang, Xianzhou Lu, Yanyan Shen, Ying Liu, Qiqing Zeng, Xianrong Liu, Wenkai Bin, Meili Li

**Affiliations:** ^1^Department of Infections, The Affiliated Nanhua Hospital, Hengyang Medical School, University of South China, Hengyang, China; ^2^Department of Hepatobiliary Surgery, The Affiliated Nanhua Hospital, Hengyang Medical School, University of South China, Hengyang, China; ^3^Department of Ultrasound Medicine, The Affiliated Nanhua Hospital, Hengyang Medical School, University of South China, Hengyang, China; ^4^Animal Injury and Poisoning Treatment Center, The Affiliated Nanhua Hospital, Hengyang Medical School, University of South China, Hengyang, China; ^5^Department of Cardiac Function, The Affiliated Nanhua Hospital, Hengyang Medical School, University of South China, Hengyang, China

**Keywords:** fatty acids, CVD, metabolomics, prediction model, SCORE2

## Abstract

**Background:**

Improving 10-year cardiovascular risk prediction beyond the established SCORE2 algorithm is a clinical need. The plasma omega-6/omega-3 (O6:O3) polyunsaturated fatty acid (PUFA) ratio, a marker of inflammatory balance, is a promising biomarker for enhancing risk stratification. We aimed to evaluate if adding the O6:O3 ratio to the SCORE2 model improves the prediction of major adverse cardiovascular events (MACE).

**Methods:**

We conducted a prospective cohort study of 183,230 UK Biobank participants (aged 50–69 years, free of baseline cardiovascular disease or diabetes). The plasma O6:O3 ratio was measured by nuclear magnetic resonance (NMR) spectroscopy. We compared the predictive performance of the SCORE2 model with and without the O6:O3 ratio in an independent validation cohort (*N* = 54,940) using Harrell’s C-index, Net Reclassification Improvement (NRI), and Integrated Discrimination Improvement (IDI).

**Results:**

In the validation set, adding the O6:O3 ratio to SCORE2 significantly increased the C-index from 0.742 (95% CI: 0.738–0.746) to 0.747 (95% CI: 0.743–0.751) (*p* < 0.001). The extended model also significantly improved risk reclassification (NRI 8.4, 95% CI: 3.6–12.2%; IDI 0.021, 95% CI: 0.010–0.032). This improvement was more pronounced in men than in women, and both models remained well-calibrated.

**Conclusion:**

Incorporating the plasma O6:O3 PUFA ratio provides a modest but statistically significant improvement in 10-year MACE risk prediction with the SCORE2 algorithm. As a modifiable biomarker, the O6:O3 ratio holds potential to refine risk stratification and guide personalized nutritional interventions.

## Introduction

Cardiovascular disease (CVD) persists as the leading cause of morbidity and mortality worldwide, accounting for nearly one-third of all global deaths annually (1, 2). A cornerstone of primary prevention is the accurate stratification of individuals according to their future risk, which enables the targeted application of preventive therapies and lifestyle interventions (3). Consequently, clinical practice guidelines heavily rely on risk prediction models that integrate established risk factors to estimate an individual’s long-term probability of experiencing a major cardiovascular event (4).

The recently developed SCORE2 algorithm represents the current state-of-the-art for estimating the 10-year risk of major adverse cardiovascular events (MACE) in European populations free of pre-existing CVD or diabetes (5). While SCORE2 provides a robust foundation for clinical risk assessment, there remains a critical need to enhance predictive precision, as a substantial “residual risk” of cardiovascular events persists even when traditional risk factors are optimally managed (6). This highlights a compelling need to identify and incorporate novel biomarkers that capture pathological pathways beyond those reflected by conventional factors.

The omega-6 to omega-3 polyunsaturated fatty-acid (PUFA) ratio is a circulating fatty-acid biomarker that reflects the balance between lipid mediators with pro-inflammatory potential (omega-6) and anti-inflammatory potential (omega-3) (7). Unlike direct inflammatory proteins such as C-reactive protein or interleukin-6, this ratio is not a cytokine marker; rather, it integrates long-term dietary exposure and endogenous metabolism into a composite measure of lipid balance relevant to atherogenesis (8, 9). Although the NMR metabolomics platform in UK Biobank quantifies more than 200 biomarkers, we selected the omega-6/omega-3 ratio *a priori* based on its strong biological rationale and prior evidence linking PUFA balance with cardiovascular outcomes (10, 11). Specifically, (i) this ratio provides a single integrative biomarker that may capture residual cardiovascular risk beyond SCORE2 covariates—including age, blood pressure, and cholesterol—given its only modest correlations with these factors; (ii) it was directly quantified with high reproducibility in UK Biobank using a standardized, high-throughput NMR platform, whereas alternative indices such as the erythrocyte Omega-3 Index were not available at scale; and (iii) previous studies consistently report that higher omega-6 and lower omega-3 status are associated with adverse cardiovascular outcomes, supporting the plausibility of this marker for incremental prediction (12).

Therefore, this study aimed to investigate whether the addition of the NMR-quantified Omega-6 to Omega-3 fatty acid ratio to the established SCORE2 algorithm improves the prediction of 10-year MACE risk in a large, prospective cohort of European adults without baseline CVD or diabetes.

## Methods

### Study population

The data for this study were sourced from the UK Biobank (UKB), a major population-based prospective cohort (13). This landmark research resource recruited over half a million men and women, aged between 40 and 69 years, from 22 assessment centers across England, Scotland, and Wales between 2006 and 2010. All participants provided informed consent, and the study received ethical approval from the North West Multi-centre Research Ethics Committee.

**Figure 1:**
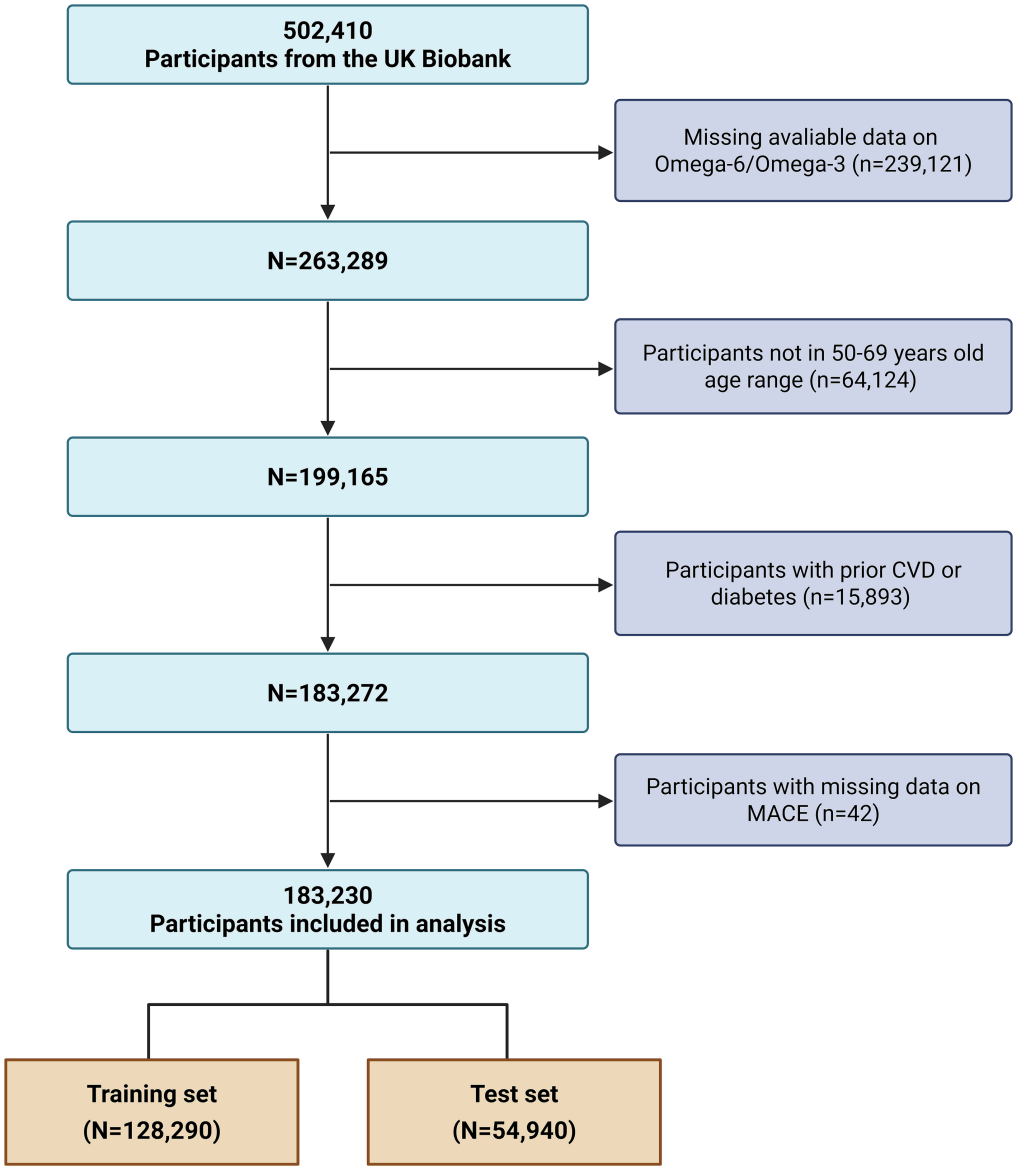
Flowchart of participant selection from the UK Biobank. The flowchart illustrates the sequential application of inclusion and exclusion criteria, starting from the total UK Biobank cohort to the final analytical sample of 183,230 participants, and the subsequent partitioning into training and validation sets. CVD, cardiovascular disease; MACE, major adverse cardiovascular events.

The selection of the final analytical cohort for the present investigation followed a multi-step, sequential exclusion process as illustrated in the study flowchart (Figure 1). From the initial UKB participants, we first identified 263,289 individuals for whom metabolomic data on the Omega-6 to Omega-3 fatty acids ratio were available. Next, we defined the age range for inclusion. While the standard SCORE2 algorithm applies to individuals aged 40–69 years (5), we restricted eligibility to participants aged 50 to 69 years at baseline (*n* = 64,124 excluded). This decision to exclude the 40–49 age range specifically aimed to mitigate the well-documented “healthy volunteer bias” within the UKB cohort (14). This bias is particularly pronounced among younger participants leading to a significantly lower prevalence of cardiovascular disease compared to the general population. Therefore, focusing on the 50–69 age range represents an approach consistent with prior studies to minimize potential bias while maintaining a large, robust sample for risk prediction analysis (15). Subsequently, we excluded 15,893 participants with a documented history of prevalent cardiovascular disease or diabetes at the time of enrollment, yielding a population of 183,272 individuals free of these conditions. A final exclusion step removed 42 individuals who had missing data on MACE outcomes.

This process yielded a final analytical cohort of 183,230 participants. For the purposes of model development and internal validation, this cohort was randomly partitioned into a training set, comprising 70% of the participants (*N* = 128,290), and a validation set, containing the remaining 30% (*N* = 54,940).

### Measurement of the omega-6 to omega-3 fatty acid ratio

Baseline non-fasting venous blood samples were collected at UKB assessment centres by trained nurses, processed within 24 h, and plasma aliquots were stored long-term at −80 °C according to standardized protocols (16). In 2019–2020, aliquots were transported on dry ice to Nightingale Health (Helsinki, Finland) for metabolomic profiling by high-throughput nuclear magnetic resonance (NMR) spectroscopy. This platform quantifies ~250 metabolic measures, including lipoproteins, fatty acids, amino acids, and glycolysis-related metabolites, with high reproducibility (17).

The NMR spectroscopy platform quantifies approximately 250 circulating biomarkers, for the present study, however, we pre-specified the plasma omega-6/omega-3 fatty acid ratio as the sole biomarker of interest, derived from directly quantified concentrations of total n-6 and n-3 PUFAs. Rigorous quality-control procedures were applied, including the use of internal calibration standards, blinded duplicate samples across analytical batches, and automated detection of outlier spectra (16). Statistical QC measures ensured consistency across the >200,000 UK Biobank samples, and the analytical coefficient of variation for fatty-acid measures was generally <5% (16).

### SCORE2 model covariates

The established risk factors that constitute the European Society of Cardiology’s SCORE2 algorithm were ascertained for all participants at their baseline assessment (5). These covariates include age, sex, current smoking status, systolic blood pressure (SBP), total cholesterol, and high-density lipoprotein cholesterol (HDL-C).

Information on age, sex, and lifestyle factors was collected via standardized, self-completed questionnaires. For the purpose of the model, smoking status was dichotomized into ‘current smoker’ or ‘non-current smoker’. Physical measurements, including SBP, were performed by trained staff; SBP was determined from automated readings using an Omron digital blood pressure monitor on the participant’s left upper arm. The concentrations of total cholesterol and HDL-C in plasma were quantified using an enzymatic method on a Beckman Coulter AU5800 clinical chemistry analyzer.

### MACE incident assessment

Participants were prospectively followed for a maximum of 10 years from their baseline assessment. The observation period for each individual concluded upon the diagnosis of a first MACE, death from a non-cardiovascular cause, or the completion of the 10-year follow-up, whichever of these occurred earliest (18). Although our primary analyses focused on 10-year risk, the actual follow-up period varied across individuals, with a median of 11.0 years (interquartile range 10.5–12.1 years). Importantly, >99% of participants had either experienced an event or contributed at least 10 years of follow-up.

The study’s primary endpoint was a composite of MACE, an outcome definition aligned with that of the original SCORE2 algorithm. A detailed definition of the MACE components is provided in Supplementary Table S1. Incident events were captured via the UK Biobank’s robust, routine linkage to national electronic health records. This comprehensive data linkage enabled the identification of non-fatal events, namely myocardial infarction and stroke, from hospital admission and primary care databases. Fatal cardiovascular events were determined through an analysis of cause-of-death information obtained from national death certificate registries.

### Statistical analyses

Baseline characteristics of the study cohort were presented as means with standard deviations (SD) for continuous variables and as counts with percentages (N, %) for categorical variables. To compare characteristics between participants who did and did not develop MACE during follow-up, independent t-tests were used for continuous variables and chi-squared (χ^2^) tests were applied for categorical variables. In addition, we compared baseline characteristics between these two subsamples to ensure representativeness (Supplementary Table S2).

Correlation analyses were conducted between the omega-6/omega-3 ratio and the individual components of the SCORE2 algorithm. Additionally, a multivariable Cox proportional hazards regression model was used to investigate the association between the omega-6/omega-3 ratio and the risk of MACE, after adjusting for all SCORE2 covariates. For supplementary analyses, we also evaluated the associations of absolute plasma concentrations of total omega-6 and total omega-3 fatty acids with MACE risk. To further explore the shape of this association, we fitted Cox models with restricted cubic splines (RCS) using four knots placed at the 5th, 35th, 65th, and 95th percentiles of the omega-6/omega-3 ratio distribution, following Harrell’s recommended approach (19). This approach allowed for a flexible modeling of the hazard ratio across the distribution of the omega-6/omega-3 ratio and included a formal test for non-linearity.

For the primary analysis, two prediction models were developed on the training set and subsequently evaluated on the independent validation set: (1) the established SCORE2 algorithm, and (2) an extended model incorporating the omega-6/omega-3 ratio as an additional predictor. The performance of these models was rigorously evaluated through a comprehensive assessment. Model discrimination, the ability to distinguish between individuals who did and did not develop MACE, was quantified using Harrell’s C-index, with the change in C-index between models being statistically tested and visualized with Receiver Operating Characteristic (ROC) curves. Furthermore, we assessed the improvement in risk stratification by calculating the Integrated Discrimination Improvement (IDI) and both continuous and category-based Net Reclassification Improvement (NRI) (20). For the categorical NRI, participants were classified into three predefined 10-year risk groups based on SCORE2 guidelines: low risk (<5%), intermediate risk (5 to <10%), and high risk (≥10%) (5). Model calibration was assessed to determine the agreement between predicted probabilities and observed outcomes. We generated a calibration plot by graphing observed event frequencies against predicted probabilities for deciles of risk in the validation cohort. To evaluate the clinical utility of the extended model, we performed decision curve analysis (DCA). The net benefit of the SCORE2 model alone was compared against the extended model (SCORE2 + O6:O3 ratio) and the default strategies of treating all or no individuals across a range of clinically relevant threshold probabilities.

All statistical analyses were conducted using R software, version 4.4.1 (R Foundation for Statistical Computing, Vienna, Austria). A small proportion of participants had missing data for one or more covariates. The extent of missingness for each variable is detailed in Supplementary Table S3. Missing values for covariates were imputed using the random forest-based *missForest* package (21). A two-sided *p*-value below 0.05 was considered statistically significant for all tests.

## Results

### Baseline characteristics

The baseline characteristics of the 183,230 participants included in the final analysis are detailed in Table 1. The mean age of the cohort was 59.8 years (SD 5.4), and a majority of the participants were female (56.5%). Current smokers constituted 9.2% of the study population. At baseline, the mean SBP was 142.4 mmHg, and the mean omega-6/omega-3 ratio for the entire cohort was 9.5.

**Table 1:** Baseline characteristics of selected participants.

Baseline characteristics	Total (*N* = 183,230)	MACE status		
		No (*N* = 169,941)	Yes (*N* = 13,289)	*p*-value
Female, *N* (%)	103,571 (56.5)	98,445 (57.9)	5,126 (38.6)	<0.001
Age (years)	59.8 (5.4)	59.6 (5.4)	62.0 (5.2)	<0.001
Current smoker, *N* (%)	16,889 (9.2)	14,680 (8.6)	2,209 (16.6)	<0.001
Systolic blood pressure (mmHg)	142.4 (19.7)	139.1 (19.6)	147.0 (20.8)	<0.001
Total cholesterol (mmol/L)	5.8 (1.1)	5.8 (1.1)	5.7 (1.2)	0.003
HDL cholesterol (mmol/L)	1.5 (0.4)	1.5 (0.4)	1.4 (0.4)	<0.001
Omega-6/Omega-3	9.5 (4.2)	9.5 (4.2)	9.9 (4.7)	<0.001

Values are expressed as mean (standard deviation) for continuous variables, and *N* (%) for categorical variables. *p*-values were calculated using *t*-tests for continuous variables and χ^2^ tests for categorical variables. HDL, high-density lipoprotein; MACE, major adverse cardiovascular event; omega-6/omega-3, omega-6 fatty acids to omega-3 fatty acids ratio; SBP, systolic blood pressure.

Over the 10-year follow-up period, 13,289 (7.3%) participants experienced a MACE. A comparison of baseline characteristics revealed multiple statistically significant differences between individuals who developed MACE and those who did not. Participants in the MACE group were, on average, older (62.0 vs. 59.6 years), less likely to be female (38.6% vs. 57.9%), more likely to be current smokers (16.6% vs. 8.6%), and exhibited higher SBP (147.0 vs. 139.1 mmHg); all with *p*-values <0.001. This group also presented with significantly lower levels of HDL-C (1.4 vs. 1.5 mmol/L, *p* < 0.001). Notably, the mean omega-6/omega-3 ratio was significantly higher among participants who later experienced a MACE compared to those who remained event-free (9.9 vs. 9.5, *p* < 0.001).

### Association of the omega-6/omega-3 ratio with MACE risk

**Figure 2:**
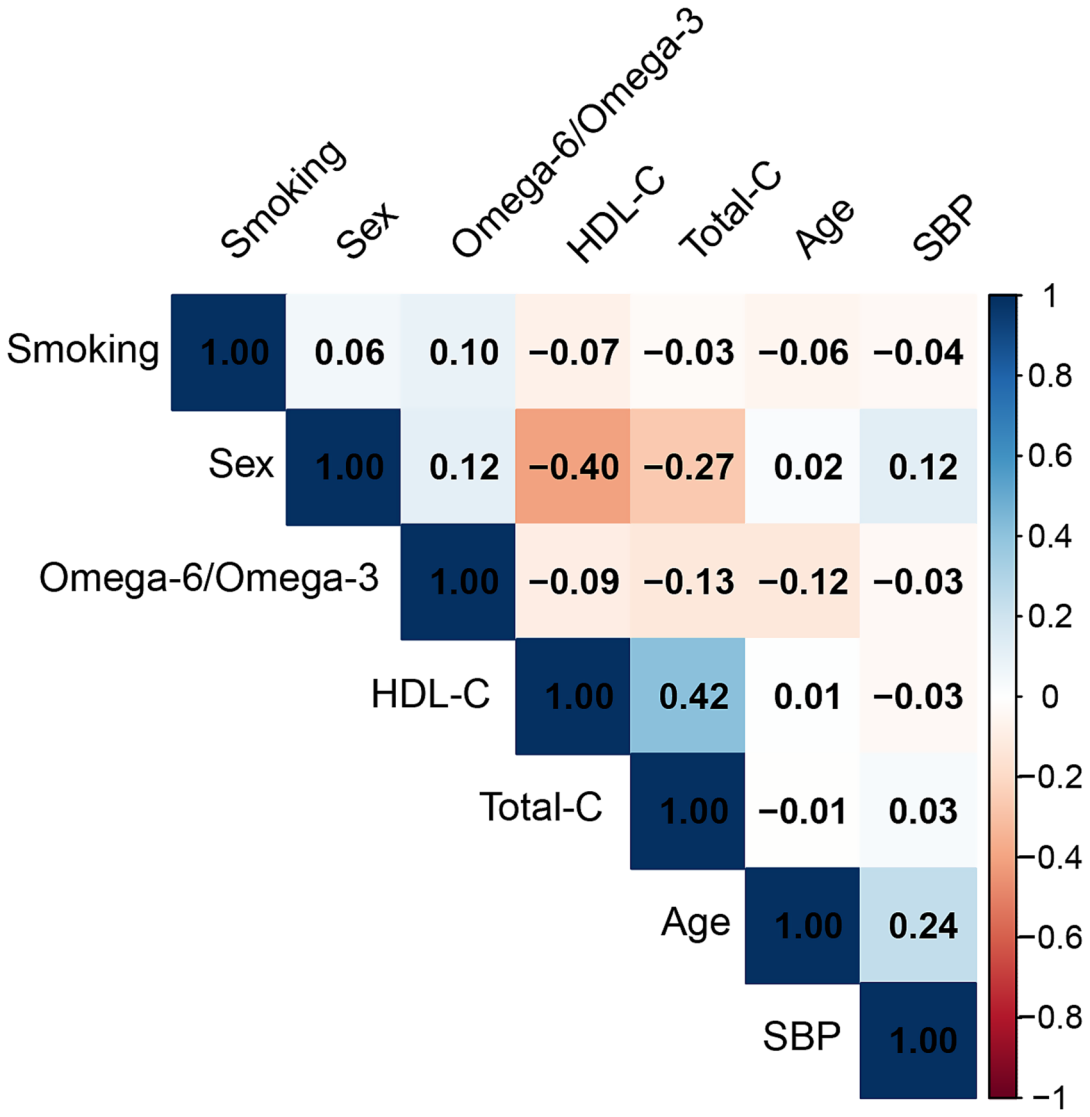
Pearson correlation matrix of the omega-6/omega-3 ratio and SCORE2 covariates. The matrix displays the correlation coefficients between the plasma omega-6/omega-3 ratio, age, sex, smoking status, systolic blood pressure (SBP), total cholesterol (total-C), and high-density lipoprotein cholesterol (HDL-C). The color scale indicates the strength and direction of the correlation, with blue representing a positive correlation and red representing a negative correlation.

The omega-6/omega-3 ratio showed only weak correlations with SCORE2 covariates (Figure 2). The strongest, albeit weak, correlations were observed with total cholesterol (r = −0.13), age (r = −0.12), and sex (r = 0.12), while its correlation with other factors like SBP was negligible (r = −0.03).

After adjustment for all SCORE2 covariates, each one-unit increase in the omega-6/omega-3 ratio was associated with an increased risk of MACE. Each one-unit increment in the ratio corresponded to a 1% increase in the hazard of MACE (HR: 1.03; 95% CI: 1.03–1.04). In supplementary analyses, higher absolute plasma omega-6 concentrations were associated with increased MACE risk (HR per SD = 1.06; 95% CI: 1.04–1.08), whereas higher omega-3 concentrations were inversely associated with MACE (HR per SD = 0.93; 95% CI: 0.91–0.95).

**Figure 3:**
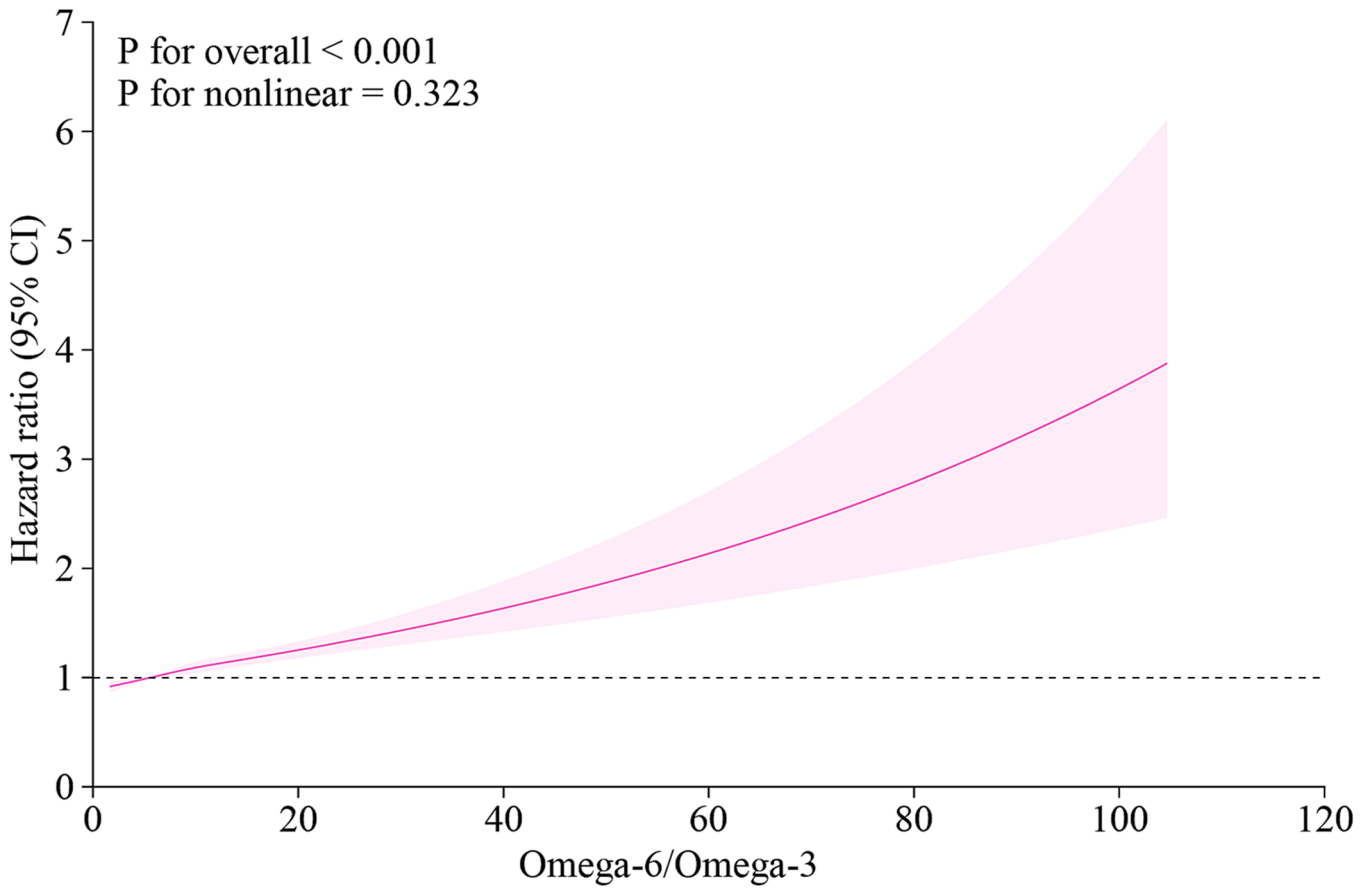
Dose–response relationship between the plasma omega-6/omega-3 ratio and the risk of MACE. The curve was plotted using a cox proportional hazards model with restricted cubic splines. The solid line represents the multivariable-adjusted hazard ratio (HR), and the shaded area indicates the 95% confidence interval (CI). The model was adjusted for age, sex, current smoking status, systolic blood pressure, total cholesterol, and HDL cholesterol. MACE, major adverse cardiovascular events; HR, hazard ratio; CI, confidence interval.

The dose–response relationship between the omega-6/omega-3 ratio and MACE risk was further visualized using restricted cubic splines (Figure 3). The analysis demonstrated a clear, monotonically increasing risk of MACE with higher levels of the ratio. This positive association was statistically significant (P for overall < 0.001), and there was no evidence of a significant deviation from linearity (P for nonlinear = 0.323).

### Improvement in cardiovascular risk prediction

**Figure 4:**
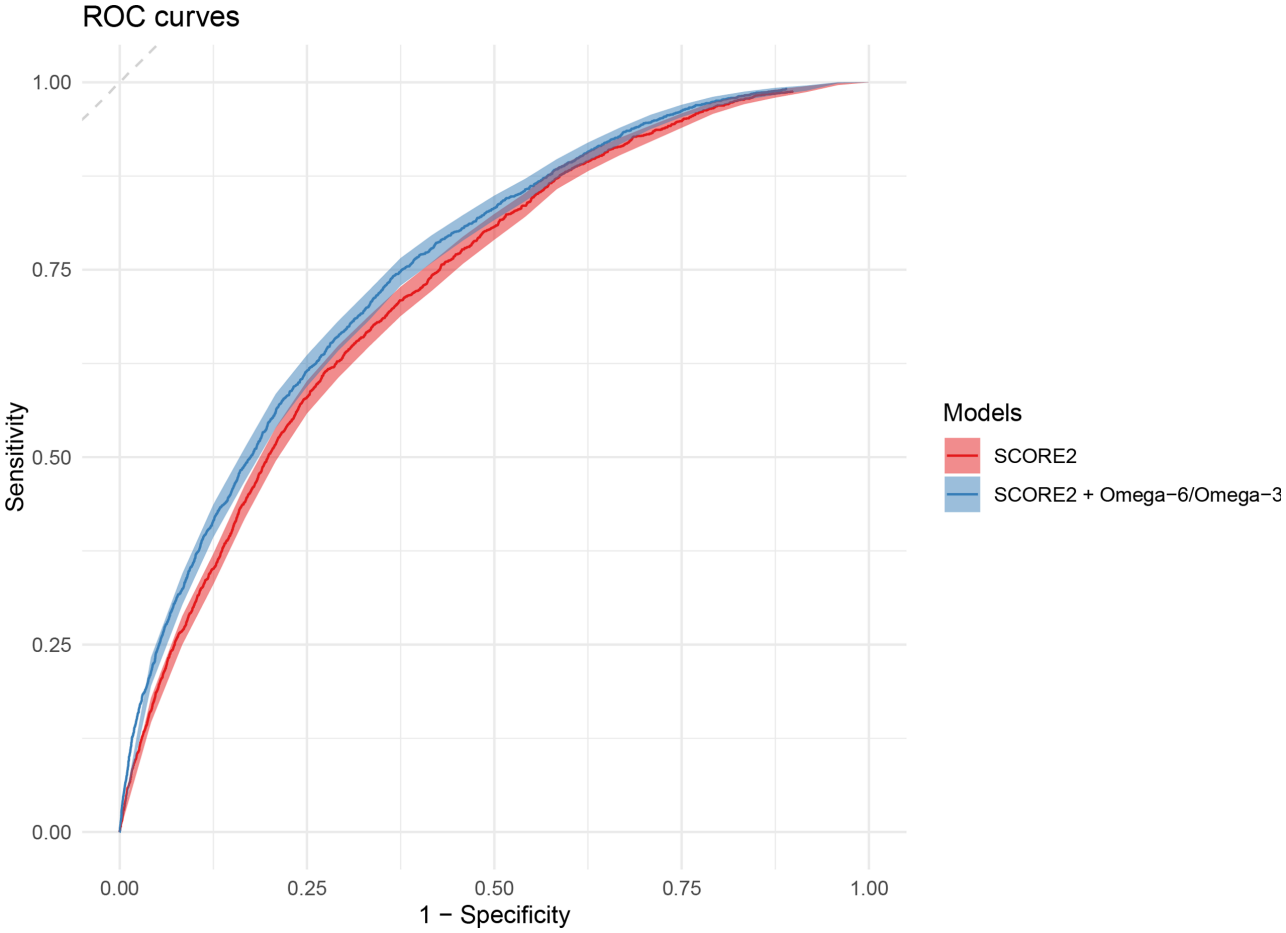
Receiver operating characteristic (ROC) curves for the prediction of 10-year MACE. The plot compares the discriminative ability of the original SCORE2 model (red) and the extended model including the omega-6/omega-3 ratio (blue) in the validation set. The diagonal dashed line represents the performance of a random classifier (area under the curve = 0.5). ROC, Receiver Operating Characteristic; MACE, major adverse cardiovascular events.

The predictive performance of the original SCORE2 model and the extended model incorporating the omega-6/omega-3 ratio was evaluated in the independent validation set, with the primary results summarized in Table 2. In the validation cohort, the addition of the omega-6/omega-3 ratio to the SCORE2 model led to a statistically significant improvement in discrimination. For the total population, the Harrell’s C-index increased from 0.742 (95% CI: 0.738, 0.746) for the original SCORE2 model to 0.747 (95% CI: 0.743, 0.751) for the extended model (P for comparison <0.001). Figure 4 shows the ROC curves for the two models. This enhancement was observed in both sexes. Among men, the C-index rose from 0.751 (95% CI: 0.745, 0.756) to 0.757 (95% CI: 0.751, 0.762) (*p* < 0.001), and among women, it increased from 0.734 (95% CI: 0.726, 0.742) to 0.737 (95% CI: 0.729, 0.750) (*p* = 0.032).

**Table 2:** Predictive performance of the SCORE2 model with omega-6/omega-3 added.

Metrics	Total	Men	Women
Training set (70% of UK Biobank, *N* = 128,290)			
C-index (SCORE2)	0.745 (0.743, 0.747)	0.751 (0.748, 0.755)	0.738 (0.733, 0.743)
C-index (+omega-6/omega-3)	0.749 (0.747, 0.752)	0.756 (0.752, 0.760)	0.742 (0.737, 0.747)
*p*-values for C-index comparison	<0.001	<0.001	<0.001
Validation set (30% of UK Biobank, *N* = 54,940)			
C-index (SCORE2)	0.742 (0.738, 0.746)	0.751 (0.745, 0.756)	0.734 (0.726, 0.742)
C-index (+omega-6/omega-3)	0.747 (0.743, 0.751)	0.757 (0.751, 0.762)	0.737 (0.729, 0.750)
*P*-values for C-index comparison	<0.001	<0.001	0.032
NRI	8.4 (3.6, 12.2)	8.9 (2.7, 15.0)	5.6 (−0.3, 11.6)
IDI	0.021 (0.010, 0.032)	0.024 (0.009, 0.038)	0.017 (0.000, 0.035)

IDI, integrated discrimination improvement; NRI, net reclassification improvement; omega-6/omega-3, omega-6 fatty acids to omega-3 fatty acids ratio.

The enhanced model also demonstrated a significant improvement in risk reclassification. For the total population, the NRI was 8.4% (95% CI: 3.6, 12.2%), and the IDI was 0.021 (95% CI: 0.010, 0.032). The improvement in risk stratification was more pronounced in men (NRI: 8.9, 95% CI: 2.7, 15.0%; IDI: 0.024, 95% CI: 0.009, 0.038) than in women (NRI: 5.6, 95% CI: −0.3, 11.6%; IDI: 0.017, 95% CI: 0.000, 0.035).

**Figure 5:**
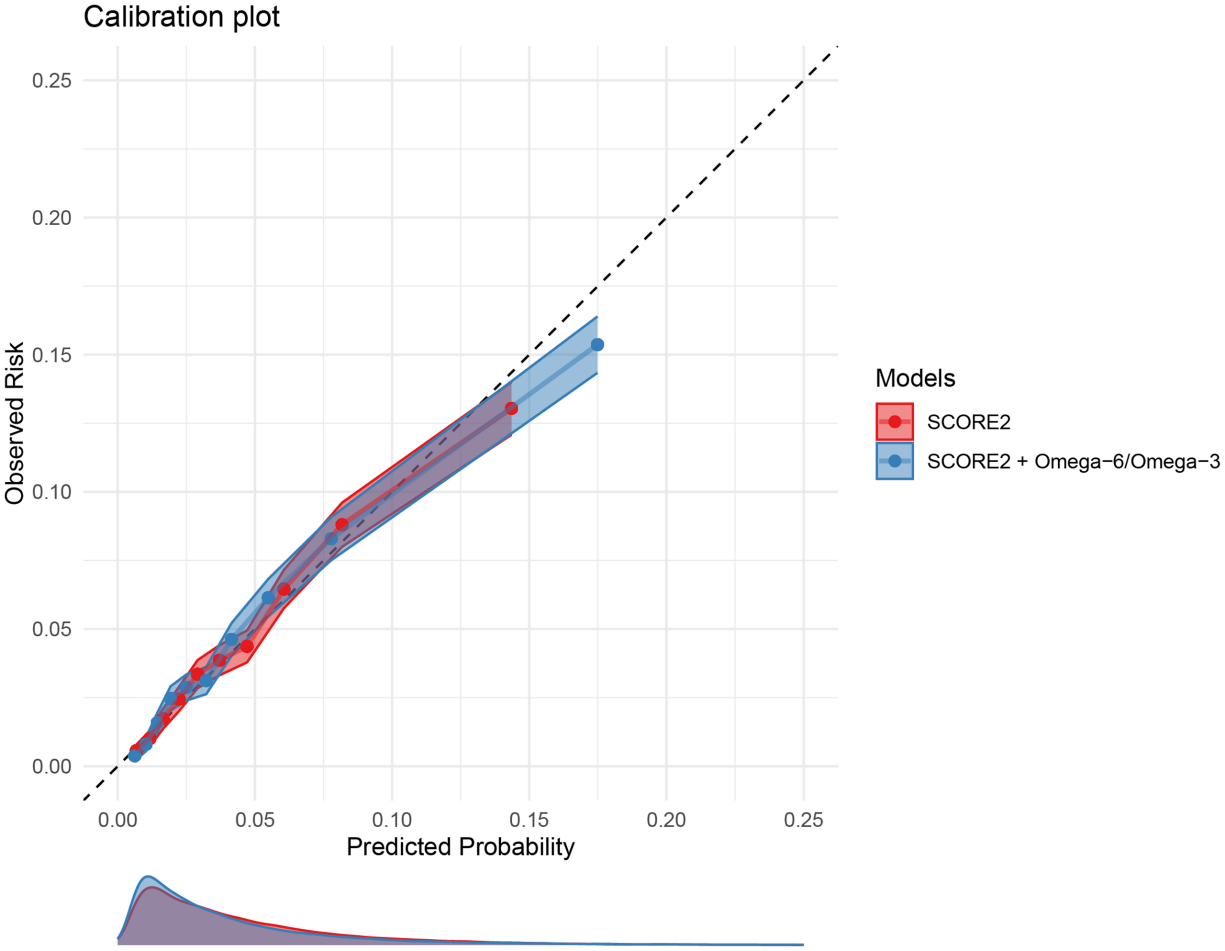
Calibration plots for the 10-year MACE risk prediction models. The plot compares the predicted 10-year MACE risk (x-axis) with the observed MACE incidence (y-axis) for the original SCORE2 model (red) and the extended model including the omega-6/omega-3 ratio (blue) in the validation set. The dashed diagonal line represents perfect calibration. Points represent deciles of predicted risk. MACE, major adverse cardiovascular events.

The calibration of both the original and extended models was assessed in the validation cohort. Figure 5 displays the calibration plots, showing the relationship between predicted probabilities and observed MACE frequencies. Both models exhibited excellent calibration, with the calibration curves for both models closely following the line of perfect calibration, indicating a strong agreement between predicted and actual risk.

To quantify the clinical significance, a decision curve analysis was performed (Supplementary Figure S1). The analysis revealed that while both models were superior to the default strategies of treating all or no patients, the extended model incorporating the O6:O3 ratio yielded a consistently higher net benefit than the SCORE2 model alone. This superiority was evident across a broad and clinically relevant range of threshold probabilities, from approximately 5 to 30%.

## Discussion

This study demonstrates that the plasma omega-6/omega-3 fatty-acid ratio, measured by high-throughput NMR metabolomics, provides incremental prognostic information for cardiovascular risk prediction beyond the SCORE2 algorithm. By analyzing more than 180,000 participants from the UK Biobank, we showed that this ratio modestly but significantly improved risk discrimination and reclassification while maintaining excellent calibration. To our knowledge, this is the first large-scale European study to evaluate the clinical utility of the omega-6/omega-3 ratio in the context of SCORE2, thereby highlighting its potential relevance as a novel biomarker for refining primary prevention strategies.

### Context with previous research

Modern Western dietary patterns are often associated with elevated omega-6 relative to omega-3 intake; however, our findings pertain to the circulating plasma omega-6/omega-3 ratio, which may reflect both dietary and metabolic influences. The clinical relevance of this biomarker lies in its potential to improve cardiovascular risk prediction when added to SCORE2, independent of its dietary or evolutionary context (22, 23). Mechanistically, an elevated ratio fosters a prothrombotic and pro-inflammatory state by favoring the overproduction of potent eicosanoids from the omega-6 precursor arachidonic acid, such as the vasoconstrictor and platelet aggregator thromboxane A2 and the inflammatory mediator leukotriene B4 (24). This biochemical framework is strongly supported by large-scale prospective cohort studies that link this imbalance to clinical outcomes (25). A landmark analysis of the UK Biobank cohort, which utilized objective plasma measurements, found that individuals in the highest quintile of the omega-6/omega-3 ratio had a 31% greater risk of cardiovascular mortality compared to those in the lowest quintile (23). Furthermore, evidence from secondary prevention trials, such as the Lyon Diet Heart Study, demonstrated that a dietary intervention achieving a lower ratio of approximately 4:1 was associated with a 70% reduction in total mortality, reinforcing the clinical importance of correcting this dietary imbalance (26).

This analysis also contributes to the ongoing effort to enhance cardiovascular risk stratification by incorporating novel fatty acid biomarkers into established prediction models. Previous research has primarily focused on the predictive value of absolute concentrations of individual fatty acids, particularly the Omega-3 Index (the sum of EPA and DHA in red blood cell membranes) (27–29). For example, one study found that adding the Omega-3 Index to the Pooled Cohort Equations (PCE) for atherosclerotic cardiovascular disease (ASCVD) prediction modestly but significantly increased the AUC (27). Similarly, other investigations have incorporated various fatty acids into broader metabolite risk scores, reporting significant increases in the C-statistic when added to models like QRISK3 for patients with type 2 diabetes (30, 31). However, these studies did not specifically evaluate the incremental predictive utility of the omega-6/omega-3 ratio, a metric which may better reflect the underlying pro-inflammatory potential. Our research addresses this gap, demonstrating that this simple, integrated ratio provides a modest but statistically significant enhancement to risk prediction beyond conventional factors, consistent in magnitude with prior investigations using individual fatty acid profiles.

### Biological mechanisms

The omega-6/omega-3 ratio provides biological plausibility for the observed associations by reflecting competing substrate flux through COX/LOX pathways: higher omega-6 favors more pro-inflammatory/pro-thrombotic eicosanoids (e.g., TXA2, LTB4), whereas higher omega-3 favors less inflammatory mediators and specialized pro-resolving mediators (SPMs) (32–39).

### Potential applications

The findings of this study suggest that the omega-6/omega-3 ratio could serve as a valuable clinical tool to enhance cardiovascular risk stratification beyond current models. Existing prediction models based on traditional risk factors often provide limited power in predicting recurrent cardiovascular events, particularly in the context of secondary prevention (40). As a modifiable biomarker, the omega-6/omega-3 ratio offers a tangible target for personalized nutritional interventions. Measuring the ratio could help clinicians identify at-risk individuals not captured by traditional metrics and subsequently monitor the biological efficacy of dietary changes aimed at restoring a more favorable fatty acid balance. This biomarker-guided approach could optimize prevention strategies, moving beyond generic advice to consume more omega-3 s towards a more nuanced strategy that also addresses the excessive consumption of omega-6 s. The well-documented inconsistency of large-scale omega-3 supplementation trials may be partly explained by a failure to account for baseline PUFA status or the high background intake of omega-6 s (23, 41). A personalized approach that targets the ratio could help clarify these conflicting results and lead to more effective prevention tools.

### Strengths and limitations

The primary strengths of this study include its large, prospective design and the use of an objective, NMR-quantified biomarker, which avoids the inaccuracies of self-reported dietary data and offers high reproducibility for large-scale studies (42). However, several limitations must be acknowledged. As an observational study, our findings demonstrate a strong association rather than definitive causality. Nevertheless, the prospective design, which establishes that the measured biomarker status precedes the clinical outcome, provides evidence that is suggestive of a causal relationship and warrants confirmation through other methodological approaches, such as Mendelian randomization. The analysis relied on a single baseline measurement, which does not account for changes in diet over the follow-up period. Furthermore, while statistically significant, the magnitude of the improvement in discrimination (ΔC = 0.005) is modest from a clinical standpoint. It is important to contextualize this result within large-scale epidemiology; in cohorts with very large sample sizes and high statistical power, predictive improvements from single biomarkers are typically incremental. Nonetheless, the practical implementation of this finding for population screening is constrained by the cost-effectiveness of NMR-based quantification. Therefore, we suggest that the primary importance of the omega-6/omega-3 ratio as a promising biomarker lies in its potential role in guiding personalized nutritional interventions, potentially in combination with other cost-effective markers, rather than in its immediate, standalone impact on risk algorithm calibration. In addition, the UK Biobank cohort is subject to a “healthy volunteer bias,” meaning participants are generally healthier, less diverse, and from higher socioeconomic backgrounds than the general population, which may limit the generalizability of our findings to more vulnerable groups who bear the greatest burden of cardiovascular disease (14). Finally, it is crucial to distinguish between the circulating plasma O6:O3 ratio measured in our study and estimates of dietary fatty acid intake. Our study utilized a plasma biomarker, which should not be directly equated with dietary consumption. Plasma fatty acid concentrations are an integrated measure, reflecting not only recent and long-term dietary patterns but also complex endogenous metabolic processes. These processes include the interconversion of fatty acids and the competitive metabolism of omega-6 and omega-3 PUFAs for the same desaturase and elongase enzymes, which can be influenced by genetic and other host factors. Therefore, while the plasma O6:O3 ratio is a powerful biomarker of internal metabolic status, it is not a pure proxy for diet. The discussions of dietary and evolutionary context within this paper are intended to provide the biological rationale for this biomarker’s potential role in health, and do not imply a direct equivalence with the measured plasma levels.

## Conclusion

In conclusion, this large-scale prospective study demonstrates that the plasma omega-6/omega-3 PUFA ratio provides incremental predictive value for 10-year MACE risk when added to the established SCORE2 algorithm. While the improvement in model discrimination is modest, the primary clinical utility of the omega-6/omega-3 ratio lies in its status as a modifiable biomarker. It has the potential to refine risk stratification and guide personalized nutritional interventions aimed at mitigating the chronic inflammatory state that drives atherosclerosis.

## Data Availability

The raw data supporting the conclusions of this article will be made available by the authors, without undue reservation.

## Glossary

AA: Arachidonic acid
ASCVD: Atherosclerotic cardiovascular disease
CI: Confidence interval
COX: Cyclooxygenase
CVD: Cardiovascular disease
DCA: Decision curve analysis
EPA: Eicosapentaenoic acid
HDL-C: High-density lipoprotein cholesterol
HR: Hazard ratio
IDI: Integrated Discrimination Improvement
LOX: Lipoxygenase
LTB4: Leukotriene B4
MACE: Major adverse cardiovascular events
NMR: Nuclear magnetic resonance
NRI: Net Reclassification Improvement
PCE: Pooled Cohort Equations
PUFA: Polyunsaturated fatty acid
RCS: Restricted cubic splines
ROC: Receiver Operating Characteristic
SBP: Systolic blood pressure
SD: Standard deviation
SPM: Specialized pro-resolving mediator
TXA2: Thromboxane A2
UKB: UK Biobank
