# Supplementary material for: The NMR-measured omega-6/omega-3 fatty acid ratio improves cardiovascular risk prediction
Source: Front Nutr. 2025 Oct 29;12:1693151. doi: 10.3389/fnut.2025.1693151 (PMC12605120; doi:10.3389/fnut.2025.1693151)
Supplement: Supplementary file 1 [file Table_1.DOCX]

**Supplementary Table S1.** Definition of endpoint major cardiovascular event (MACE)

| **Fatal MACE – cause-specific mortality due to any of the following:** | |
| --- | --- |
| *Endpoints included* | *ICD10-codes* |
| Hypertensive disease | I10-16 |
| Ischemic heart disease | I20-25 |
| Arrhythmias, heart failure | I46-52 |
| Cerebrovascular disease | I60-69 |
| Atherosclerosis/aortic aneurysm | I70-73 |
| Sudden death and death within 24 hours of symptom onset | R96.0-96.1 |
|  |  |
| *Endpoints excluded from the above endpoint:* | *ICD10-codes* |
| Myocarditis, unspecified | I51.4 |
| Subarachnoid haemorrhage | I60 |
| Subdural hemorrhage | I62 |
| Cerebral aneurysm | I67.1 |
| Cerebral arteritis | I68.2 |
| Moyamoya | I67.5 |
|  |  |
| **Non-fatal MACE** | *ICD10-codes* |
| Non-fatal myocardial infarction | I21-I23 |
| Non-fatal stroke | I61, I63-I66, I6 |
