# Supplementary material for: The NMR-measured omega-6/omega-3 fatty acid ratio improves cardiovascular risk prediction
Source: Front Nutr. 2025 Oct 29;12:1693151. doi: 10.3389/fnut.2025.1693151 (PMC12605120; doi:10.3389/fnut.2025.1693151)
Supplement: Supplementary file 2 [file Table_2.DOCX]

**Supplementary Table S2.** Baseline characteristics of participants in the training and validation cohorts

| **Baseline characteristics** | **Training set (n=128,261)** | **Validation set (n=54,969)** | **P-value** |
| --- | --- | --- | --- |
| Female, n (%) | 72,500 (56.5) | 31,071 (56.5) | 0.99 |
| Age, years (mean ± SD) | 59.8 ± 5.4 | 59.7 ± 5.5 | 0.62 |
| Current smoker, n (%) | 11,859 (9.3) | 5,030 (9.2) | 0.71 |
| SBP, mmHg (mean ± SD) | 142.3 ± 19.6 | 142.5 ± 19.7 | 0.39 |
| Total cholesterol, mmol/L (mean ± SD) | 5.8 ± 1.1 | 5.8 ± 1.1 | 0.84 |
| HDL cholesterol, mmol/L (mean ± SD) | 1.5 ± 0.4 | 1.5 ± 0.4 | 0.93 |
| Omega-6/Omega-3 ratio (mean ± SD) | 9.5 ± 4.2 | 9.5 ± 4.3 | 0.76 |
