# Supplementary material for: The NMR-measured omega-6/omega-3 fatty acid ratio improves cardiovascular risk prediction
Source: Front Nutr. 2025 Oct 29;12:1693151. doi: 10.3389/fnut.2025.1693151 (PMC12605120; doi:10.3389/fnut.2025.1693151)
Supplement: Supplementary file 3 [file Table_3.DOCX]

**Supplementary Table S3.** Summary of Missing Data for Covariates Before Imputation in the Full Cohort

| **Variable** | **N (Non-Missing)** | **N (Missing)** | **% Missing** |
| --- | --- | --- | --- |
| Age | 183,230 | 0 | 0.00% |
| Sex | 183,230 | 0 | 0.00% |
| Current Smoker | 183,105 | 125 | 0.07% |
| Systolic Blood Pressure (SBP) | 182,540 | 690 | 0.38% |
| Total Cholesterol | 181,988 | 1,242 | 0.68% |
| HDL Cholesterol | 181,975 | 1,255 | 0.69% |
| Omega-6/Omega-3 Ratio | 183,230 | 0 | 0.00% |
