# Supplementary figures and images for: The NMR-measured omega-6/omega-3 fatty acid ratio improves cardiovascular risk prediction
Source: Front Nutr. 2025 Oct 29;12:1693151. doi: 10.3389/fnut.2025.1693151 (PMC12605120; doi:10.3389/fnut.2025.1693151)

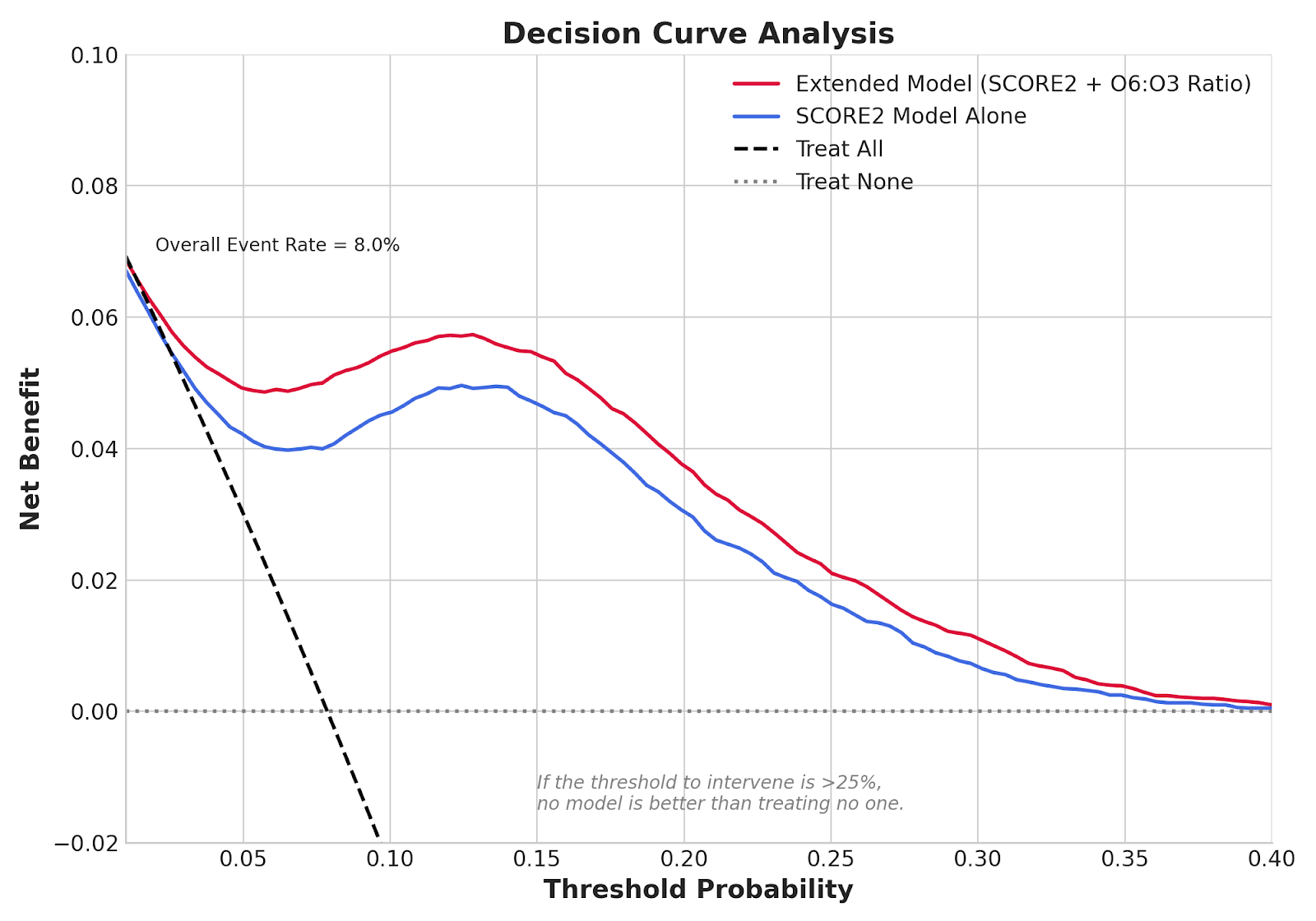

Supplement: Supplementary file 4 [file Image_1.PNG]
